# Effective methods and framework for energy-based local learning of deep neural networks

**DOI:** 10.3389/frai.2025.1605706

**Published:** 2025-08-26

**Authors:** Haibo Chen, Bangcheng Yang, Fucun He, Fei Zhou, Shuai Chen, Chunpeng Wu, Fan Li, Yansong Chua

**Affiliations:** ^1^China Nanhu Academy of Electronics and Information Technology, Jiaxing, China; ^2^China Electric Power Research Institute, Beijing, China; ^3^State Grid Shanghai Municipal Electric Power Company, Shanghai, China

**Keywords:** artificial neural network, biologically plausible learning rule, local learning, energy-based model, predictive coding

## Abstract

From a neuroscience perspective, artificial neural networks are regarded as abstract models of biological neurons, yet they rely on biologically implausible backpropagation for training. Energy-based models represent a class of brain-inspired learning frameworks that adjust system states by minimizing an energy function. Predictive coding (PC), a theoretical model within energy-based models, constructs its energy function from forward prediction errors, with optimization achieved by minimizing local layered errors. Owing to its local plasticity, PC emerges as the most promising alternative to backpropagation. However, PC face gradient explosion and vanishing challenges in deep networks with multiple layers. Gradient explosion occurs when layer-wise prediction errors are excessively large, while gradient vanishing arises when they are excessively small. To address these challenges, we propose bidirectional energy to stabilize prediction errors and mitigate gradient explosion, while using skip connections to resolve gradient vanishing problems. We also introduce a layer-adaptive learning rate (LALR) to enhance training efficiency. Our model achieves accuracies of 99.22% on MNIST, 93.78% on CIFAR-10, 83.96% on CIFAR-100, and 73.35% on Tiny ImageNet, comparable to the performance of identically structed networks trained with backprop. Finally, we developed a Jax-based framework for efficient training of energy-based models, reducing training time by half compared to PyTorch.

## 1 Introduction

Artificial neural networks (ANNs) trained using backpropagation (backprop) have achieved remarkable advancements over the past decade. Despite this success, neuroscientists have questioned the biological plausibility of backprop. A key criticism is that biological neurons adhere to rules of accessing information only from adjacent neurons locally, whereas backprop transmits information from distant neurons layer by layer via the chain rule (Crick, 1989; Stork, 1989). This disparity has prompted researchers to explore alternative solutions based on biology, particularly bio-inspired models and learning algorithms. Some studies on the structure and function of biological neural networks. Such as the simulation modeling of neural network connection methods (Yang et al., 2021; Hoffmann et al., 2024), the topological structure and interaction of neural connections in biological neural networks (Znaidi et al., 2023; Boccato et al., 2024; Salova and Kovács, 2025; Xiao et al., 2021), the interconnection structure, self-organization and self-optimization characteristics of brain-derived neurons (Yin et al., 2020), all of these studies have pointed out that the local interactions of the connections in biological neural networks have adaptive adjustment characteristics, which can optimize the overall information transmission efficiency. At the same time, some “backprop-free” local learning methods that avoid global gradient transmission have been proposed. These methods aim to modify the weights of the dynamical equations by using locally available information. Such methods are usually strongly inspired by biological synaptic plasticity and give rise to various algorithms and models. These models include self-organizing maps (Khacef et al., 2019; Hirani et al., 2024; Sa-Couto and Wichert, 2023), hebbian learning (Pogodin and Latham, 2020; Krotov and Hopfield, 2019; Moraitis et al., 2022), forward-forward algorithms (Hinton, 2022; Momeni et al., 2023), feedback alignment algorithms (Lillicrap et al., 2016; Nøkland, 2016), local error-driven (Cheng et al., 2024; Yin et al., 2023), energy-based local learning models (Bengio and Fischer, 2015; Hopfield, 1982; Scellier and Bengio, 2017; Xie and Seung, 2003).

Energy-based local learning models (EBLL) originate from the broader category of energy models, which view learning and inference as the minimization of an energy function defined over the states of model variables (such as inputs, outputs, or hidden states). These models must define and estimate an explicit global energy function. EBLL typically adheres to classical energy theories, such as the free energy principle or hopfield energy. During the energy minimization process, EBLL minimizes local energy through a locality principle, either hierarchically or in blocks, thereby avoiding the propagation of global energy gradients. Even under the guidance of classical energy theories, defining and estimating an energy function remains challenging in practical applications, such as hierarchical predictive coding (HPC) models (Friston, 2005; Friston and Stephan, 2007; Whittington and Bogacz, 2017; Buckley et al., 2017; Millidge et al., 2021) based on the free energy principle. The free energy principle (Friston, 2005; Friston et al., 2006; Friston and Stephan, 2007; Friston, 2010) is a normative theoretical framework that asserts that systems maintain a generative model and minimize a quantity called free energy to reduce the mismatch between predicted and observed sensory data. HPC (Rao and Ballard, 1999; Friston, 2003; Clark, 2013) is an implementation model that describes how the brain achieves perception through the minimization of local errors. After the free energy principle was proposed, predictive coding became an approximate implementation of it (Buckley et al., 2017; Millidge et al., 2021). In the free energy principle, predictive coding assumes the generative model to be a hierarchical Gaussian probabilistic model, and free energy is defined as the difference between an approximate variational posterior distribution and the true posterior distribution, which is not easy to estimate directly (Friston and Kiebel, 2009; Spratling, 2017; Piekarski, 2023). In most specific supervised task implementations (Whittington and Bogacz, 2017; Dold et al., 2019; Rosenbaum, 2022; Millidge et al., 2022), this expression of free energy is approximated as the sum of squared local feedforward prediction errors between layers, that is, a quadratic energy function of the squared prediction errors in a single direction. This approximate expression relies on the assumption of a Gaussian distribution. This quadratic energy function offers notable computational advantages and is widely adopted in practice. Mathematically, it is a convex function with continuous gradients and analytical derivatives, usually ensuring the existence of a unique minimum. This characteristic is particularly convenient for calculation in the process of minimizing the energy function. However, it also has significant limitations. The real perception mappings and deep network representations are often highly non-Gaussian and nonlinear. In high-dimensional spaces, this can result in substantial errors, potentially leading to instability or even divergence during the learning process. In deep networks, this manifests as gradient explosion and vanishing gradient phenomena. When the prediction error of a single internal layer in an artificial neural network is too large, it amplifies in deeper layers, leading to high energy levels and gradient explosion during the energy minimization phase. Conversely, overly small energy levels, typically caused by network depth, can impede progress toward energy minimization.

Recent studies (Pinchetti et al., 2022; Kinghorn et al., 2022) have demonstrated that HPC suffers from performance degradation or outright collapse during training when applied to complex or deep neural network architectures. To address these challenges, some approaches have been proposed. Both Kinghorn et al. (2022) and Millidge et al. (2023) introduce the weight-regularization, a regularization method for HPC that uses the L1 weight norm and a simple weight restriction strategy to prevent performance degradation. While regularization is generally an effective technique for improving stability, its practical reliability remains inconsistent. Pinchetti et al. (2022) extended the HPC based on Gaussian distribution to any probability distribution and successfully applied it to a transformer network with a single head and 128 dimensions. The energy function shifts from minimizing the numerical prediction error to minimizing the discrepancy between the predicted distribution and the true distribution, typically measured by the Kullback-Leibler (KL) divergence. However, this approach can be computationally demanding, as the KL divergence is often intractable. In many tasks, the true distribution is either unknown or cannot be analytically integrated. Therefore, this method underscores its broad applicability to any family of distributions for which an explicit analytical form of the KL divergence can be derived.

Taking into account the computational advantages of the energy minimization process, we still followed the energy function in the form of prediction error under the assumption of Gaussian distribution. However, we made a structured improvement to the energy function to overcome the gradient explosion and vanishing during training. This improvement was inspired by two key aspects in biology. First, we consider the biological perspective, focusing on the reciprocal interactions between feedforward and feedback connections in cortical regions (Rockland, 2022; Angelucci and Petreanu, 2023). Second, we take inspiration from machine learning's emulation of biological processes. For example, Lillicrap et al. (2020) suggested that feedback pathways primarily adjust neural activities to transmit information essential for effective multilayer learning. Consequently, Lillicrap et al. (2016) introduced feedback alignment (FA), a biologically plausible learning model. However, the use of fixed random feedback weights limits FA's effectiveness in deeper networks. Later studies extended FA through bidirectional learning, refining the reverse pathways to improve feedback transmission (Amit, 2019; Luo et al., 2017). Similarly, target propagation (Bengio, 2014; Lee et al., 2015) algorithm employs stacked auto-encoders to reverse reconstruct local representations, guiding the learning process. These prior research findings inspire the use of bidirectional free energy to mitigate gradient explosion issues in deep PC networks. Bidirectional energy functions go through the following mechanisms:

i) Feedback connections relay signals from higher-level to lower-level units, where they are processed to produce prediction errors.
ii) The feedback and feedforward prediction errors together create a bidirectional symmetry in energy.
iii) During the energy minimization process, the interplay between bidirectional prediction errors in both directions stabilizes neuronal updates, alleviating the issues of gradient explosion in HPC networks.

The remainder of this paper is organized as follows. Section 2 reviews HPC and its gradient explosion and vanishing problems. Section 3 presents an overview of bidirectional PC (BiPC), illustrated with a biologically inspired ANN model. This approach integrates both bottom-up and top-down predictions in each network block, ensuring stable gradient updates during energy minimization. Section 4 explores the layer-wise weight update mechanism of EBLL and introduces the layer-adaptive learning rate (LALR). By dynamically adjusting learning parameters across network layers, LALR enhances convergence speed while ensuring stability. Section 5 presents a unified energy function framework for PC and EP in a supervised learning context. We propose that the energy function under the supervised learning scenario is split into internal and external energy components. The internal energy reflects the intrinsic dynamics of the PC, while the external energy represents the impact of the loss function. We then implement a Jax-based framework (Frostig et al., 2018; Bradbury et al., 2018) for training energy-based models, reducing training time by half compared to PyTorch. Finally, Section 6 demonstrates the effectiveness of our approach through experiments, including image classification on MNIST, CIFAR10, CIFAR100, and Tiny ImageNet. The results show that our framework enables reliable learning within deep ANNs using EBLL, achieving accuracy similar to that of backprop under identical ANN conditions.

## 2 Hierarchical predictive coding

The classical HPC model in the visual cortex is an unsupervised learning framework (Rao and Ballard, 1999), where top-down processing generates predictions, with feedback pathways transmitting predictions from the activities of higher-level to lower-level units. Prediction errors are processed bottom-up, with the feedforward pathways carrying the residuals between the predictions and the actual activities (Rao and Ballard, 1999). HPC efficiently encodes input data by iteratively looping and locally predicting and correcting input signals through its hierarchical structure. However, applied to supervised machine learning tasks in an ANN, HPC functions in a manner contrary to its theoretical model described above (Millidge et al., 2022; Rosenbaum, 2022). The data *X* is assigned to layer 0 at the bottom, and labels *Y* are assigned to layer *L*+1 at the top. Predictions are made through the feedforward process, while prediction errors at each level adjust unit activities and parameters in a backward direction. The detailed process is outlined as follows.

We consider an *L*-layers feedforward network with unit states *V*, where *v*_*i*_ ∈ *V* denotes the latent states of the *ith* layer, with *v*_0_ = *X* and *v*_*L*+1_ = *Y*. We refer to Rosenbaum (2022) for the state initialization protocol, where the initial forward pass is used as initial values. In a supervised learning context, the output of the network must converge to the label *Y*. The prediction errors for each layer are defined as follows


(1) $$\begin{array}{l} \epsilon_{i}=v_{i}-{\hat{v}}_{i}\\ \;=v_{i}-g_{i}\left(v_{i-1};\theta_{i}\right), \end{array}$$

where *g*_*i*_(*v*_*i*−1_; θ_*i*_) implies the *ith* layer function applied to *v*_*i*−1_, yielding the feedforward prediction $\hat{v_{i}}=g_{i}\left(v_{i-1};\theta_{i}\right)$.

Ultimately, supervised learning HPC optimizes a global energy function *F*, which includes internal layer-wise prediction errors and a loss function applied to the set of output units (Dold et al., 2019).


(2) $$\begin{array}{l} F=\overset{L}{\underset{i=1}{\sum }}\epsilon_{i}^{2}+C, \end{array}$$

where *C* indicates the mean squared error between the output prediction and target behavior (Dold et al., 2019). Both unit states and parameter dynamics of the network can be derived as a gradient descent on the energy function *F*. Therefore, *F* can also be interpreted as the global objective function of the network (Millidge et al., 2022).

At each iteration, the network states are updated as follows: *v*_*i*_ = *v*_*i*_−η_*v*_*dv*_*i*_, where η_*v*_ refers to the step rate for *v*, and *dv*_*i*_ is expressed as:


(3) $$\begin{array}{l} dv_{i}=\frac{\partial F}{\partial v_{i}}\\ \;=\epsilon_{i}-\epsilon_{i+1}\frac{\partial g_{i+1}\left(v_{i};\theta_{i+1}\right)}{\partial v_{i}}, \end{array}$$

After sufficient iterations, *F* eventually converges to its equilibrium point *F*_*min*_. At this point, the parameters θ_*i*_ are updated as θ_*i*_ = θ_*i*_−η_θ_*dθ*_*i*_, where η_θ_ implies the step size for updating θ. The formula for *dθ*_*i*_ is given by:


(4) $$\begin{array}{l} d\theta_{i}=\frac{\partial F_{min}}{\partial \theta_{i}}\\ \;=-\epsilon_{i}\frac{\partial g_{i}\left(v_{i-1};\theta_{i}\right)}{\partial \theta_{i}}, \end{array}$$

Although supervised HPC adheres to local updates, some studies suggest that HPC in supervised learning approximates backprop. This indicates that HPC possesses notable potential. However, it also faces gradient explosion and vanishing. Gradient explosion occurs when a large ϵ_*i*_ amplifies subsequent errors. In the process of minimizing the energy function, the computation of the *dv*_*i*_, which is derived from Equation 3, depends on two terms: the first term ϵ_*i*_, and the second term $\epsilon_{i+1}\frac{\partial g_{i+1}\left(v_{i};\theta_{i+1}\right)}{\partial v_{i}}$. if ϵ_*i*_ is excessively large, even a moderate second term may still result in an overly large gradient. Furthermore, updating the state *v*_*i*_, expressed as *v*_*i*_ = *v*_*i*_−η_*v*_*dv*_*i*_, can lead to significant changes in *v*_*i*_ when $\frac{\partial F}{\partial v_{i}}$ or the learning rate η_*v*_ is excessively large. As the hierarchical network propagates forward, such drastic changes in *v*_*i*_ influence the prediction of the next layer, given by ${\hat{v}}_{i+1}=g_{i+1}\left(v_{i};\theta_{i+1}\right)$, consequently increasing ϵ_*i*+1_. This effect becomes amplified in deep networks. In cases where the network depth is substantial, errors and gradients accumulate progressively during inter-layer propagation, ultimately leading to gradient explosion. Conversely, the same mechanism can contribute to gradient vanishing.

## 3 Bidirectional predictive coding

Here, we introduce a novel, biologically plausible BiPC model. As described in Section 2, supervised HPC relies only on feedforward prediction error driven. When the prediction error in one layer is excessively large or small, the cumulative effect of forward propagation through the hierarchical network frequently results in severe explosion or vanishing of the gradient, thereby constraining its effectiveness for complex tasks. The BiPC model overcomes these limitations by incorporating bidirectional error propagation. As noted in Section 1, this bidirectional architecture is inspired by the reciprocal connectivity observed in cortical neural networks, exemplified by the interplay between feedforward pathways from the primary visual cortex (e.g., V1) to extrastriate cortex (e.g., V2, V3, V4) and feedback pathways from extrastriate cortex back to V1. These feedback signals modulate feedforward inputs–either amplifying or suppressing them–to refine visual perception. Grounded in a biologically inspired artificial neural network (ANN), the BiPC model features a feedback prediction pathway from higher to lower representations, enabling symmetric modulation between feedforward and feedback prediction errors at the same level. This symmetry suppresses excessive error signals, mitigating gradient explosion. Furthermore, skip connections are employed to strengthen the flow of gradients to deeper layers, addressing gradient vanishing and ensuring robust learning across the network.

We model a network of nodes and edges designed to simulate the cortical structure. Each node represents a cortical area in the brain, such as V1 or V2, and encodes the activation values of all units at a given time, which are subsequently transmitted to the next node through edges. The bottom node captures the initial input data features, while the top node encodes advanced features for label prediction.

The edges between these nodes represent four distinct connectivity patterns found in cortical regions: feedforward connection and feedback connection, skip connection, and recurrent connection. These connections work together to generate state predictions within cortical regions:

- The bottom-up feedforward based on inference prediction circuit extracts high-level representations from input signals for decision-making.
- The top-down feedback based on the generative prediction circuit generates estimates grounded on high-level representations from the inference circuit.
- The recurrent connections enable bidirectional propagation of node states at each level, from left to right and vice versa.

### 3.1 Feedforward prediction

We demonstrate the inference and learning process of the BiPC model using the states of an intermediate node *v*_*m, t*_ as a representative example.

**Figure 1:**
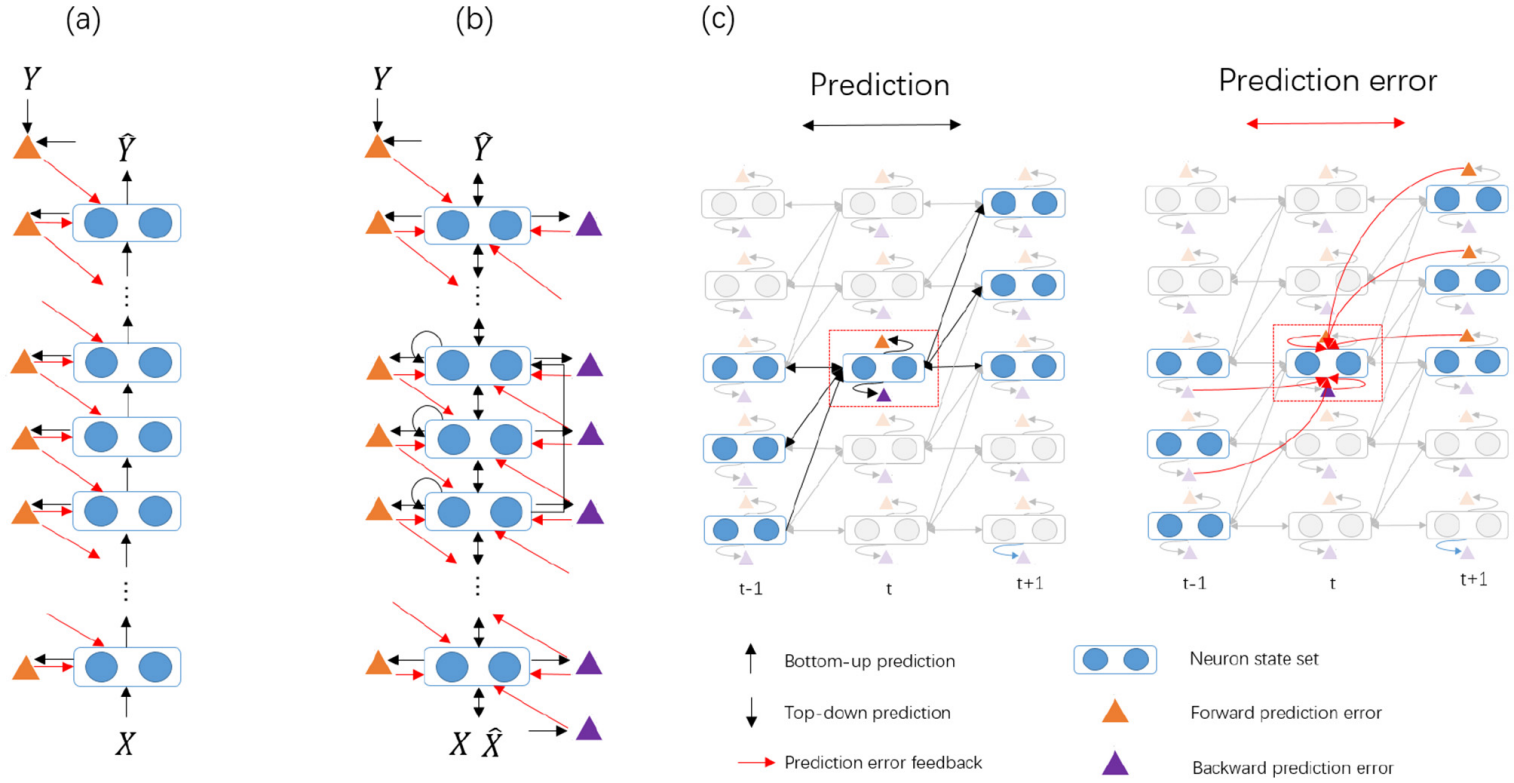
The comparison between the traditional HPC and our proposed BiPC model for supervised tasks. **(a)** In the HPC model, only bottom-up predictions (represented by black arrows) are present. Forward prediction errors (denoted by orange triangles) are computed by comparing these predictions with the actual unit states, and the derived error signals serve as feedback (shown as red arrows) to modify the unit states. **(b)** The BiPC model features symmetric connectivity, integrating both feedforward and feedback prediction routes. This results in the generation of both feedforward and feedback prediction errors (illustrated by orange and purple triangles, respectively), along with the inclusion of recurrent and skip connections. **(c)** An in-depth depiction of the evolution of unit states over time. The red dashed box highlights the unit state *v*_*m, t*_, while the semi-transparent modules represent components not directly linked to *v*_*m, t*_. On the left, both feedforward and feedback predictions for *v*_*m, t*_ are presented, producing feedforward prediction errors $\epsilon_{m,t}^{f}$ (orange triangle) and feedback prediction errors $\epsilon_{m,t}^{b}$ (purple triangle). On the right, the energy minimization phase is demonstrated, where the bidirectional prediction errors are employed to modify the unit states.

As shown in Figure 1, the feedforward state predictions for the *mth* node require three input components: the first component is the output of the node states *v*_*m*−1, *t*−1_ from the previous node processed through the feedforward connection; the second component is the output of the node states *v*_*m*−2, *t*−1_ from a lower node, processed through the skip connection; and the third component is the node's own output from the previous time step.

Let ${\hat{v}}_{m,t}^{f}$ signifies the feedforward state predictions of the *mth* node at time *t* which is expressed as:


(5) $$\begin{array}{l} {\hat{v}}_{m,t}^{f}=g_{m,m}(\overset{\text{bottom-up connection}}{\overset{︷}{g_{m-1,m}(v_{m-1,t-1};\theta_{m-1,m}}}\\ \;+\overset{\text{skip connection}}{\overset{︷}{g_{m-2,m}(v_{m-2,t-1};\theta_{m-2,m}}}\\ \;+\overset{\text{self-excitation}}{\overset{︷}{v_{m,t-1}}};\theta_{m,m}), \end{array}$$

where *g*_*m*−1, *m*_ indicates the connection function from the lower node *m*−1 to the higher node *m*, *g*_*m, m*_ implies the recurrent connection function for the node *m*, and *g*_*m*−2, *m*_ denotes a skip-connection function from the lower node *m*−2 to the higher-level node *m*. The predicted state is then compared to the actual activity of *b*_*m*_ at time step *t*, yielding the forward prediction errors, $\epsilon_{m,t}^{f}$.


(6) $$\begin{array}{l} \epsilon_{m.t}^{f}=v_{m,t}-{\hat{v}}_{m,t}^{f}, \end{array}$$

### 3.2 Feedback prediction

Previous research has examined some methods for modeling the feedback pathways to simulate the brain feedback. One approach involves creating a secondary feedback network (Hinton, 2003; Xie and Seung, 2003), often requiring the presence of reverse connections that mirror the forward connections. In this study, we design the feedback pathways to produce feedback predictions of node states, which are integrated with feedforward prediction errors to adjust these states. This process demands symmetry between the feedback and forward prediction errors. Consider *mth* node as an example, according to the principle of symmetry, there should be three feedback connections symmetrical to each feedforward, skip, and recurrent connection. We exclude higher-to-lower-level skip connections due to significant information loss during the transfer, which impedes the accurate recovery of lower-level representations. Two branches are used to generate the feedback state prediction comprising ${\hat{v}}_{m,t}^{b}$. One branch connects *v*_*m*+1, *t*+1_ to *v*_*m, t*_, with its feedback prediction comprising ${\hat{v}}_{m+1,t}^{b}$ and ${\hat{v}}_{m,t}^{b}$. The feedback prediction representation ${\hat{v}}_{m,t}^{b}$ and its errors $\epsilon_{m,t}^{b}$ are defined as follows:


(7) $$\begin{array}{l} {\hat{v}}_{m,t}^{b}=\frac{1}{2}\overset{\text{top-down connection}}{\overset{︷}{z_{m+1,m}\left(v_{m+1,t+1};\theta_{m,m+1}^{⊤}\right)}}+\frac{1}{2}\overset{\text{recurrent connection}}{\overset{︷}{z_{m,m}\left(v_{m,t+1};\theta_{m,m}^{⊤}\right)}}, \end{array}$$

(8) $$\begin{array}{l} \epsilon_{m,t}^{b}=v_{m,t}-{\hat{v}}_{m,t}^{b}, \end{array}$$

where *z*_*m*+1, *m*_ refers to the feedback function from the high-level node *m*+1 to low-level node *m*, and *z*_*m, m*_ indicates its self-recurrent feedback function. In supervised ANN tasks, the feedforward function typically handles feature extraction and downsampling operations, while the feedback function manages signal reconstruction and upsampling. The forward encoding uses convolutional operations, while the reverse decoding employs transposed convolutions. The feedback function applies transposed convolutions with the transpose of the feedforward parameters.

### 3.3 Enery function

The energy equation can be expressed in Equation 9, representing the sum of two distinct PC components–the feedforward and the feedback pathways:


(9) $$\begin{array}{l} F=\overset{T}{\underset{t=0}{\sum }}\overset{M}{\underset{m=1}{\sum }}{\left(\epsilon_{m,t}^{f}\right)}^{2}+{\left(\epsilon_{m,t}^{b}\right)}^{2}+C^{f}+C^{b}, \end{array}$$

where *C*^*f*^ indicates the feedforward loss function, specifically using cross-entropy loss for the supervised discrimination task, and *C*^*b*^ signifies the feedback loss function, employing mean squared error loss.

However, we found that this formulation often causes gradient explosion in deep networks. Since both feedforward and feedback prediction errors are squared and positive, large errors in one are not sufficiently controlled by the other, leading to a potential gradient explosion.

Therefore, we propose a revised energy function, which will be applied consistently throughout this study:


(10) $$\begin{array}{l} F=\overset{T}{\underset{t=0}{\sum }}\overset{M}{\underset{m=1}{\sum }}{\left(\epsilon_{m,t}^{f}+\epsilon_{m,t}^{b}\right)}^{2}+C^{f}+C^{b}, \end{array}$$

Equation 10 mitigates gradient explosion by balancing positive and negative cancellation of feedforward and feedback prediction errors.

With the energy function established, the node states are updated based on the energy function using the following formula. First, the states *v* are updated via gradient descent based on the energy function *F*. Subsequently, parameter updates are performed at the minimum energy *F*_min_. Both error directions are used for updating states and parameters to maintain stability during the inference and learning process, as shown in Equation 10:


(11) $$\begin{array}{l} v_{m,t}=v_{m,t}-\eta_{v}\frac{\partial F}{\partial v_{m,t}}, \end{array}$$

(12) $$\begin{array}{l} \theta_{m,i}=\theta_{m,i}-\eta_{\theta }\frac{\partial F_{\text{min}}}{\partial \theta_{m,i}}, \end{array}$$

where η_*v*_ and η_θ_ refer to the step size for states and parameters update, respectively, and θ_*m, i*_ implies the parameters from node *m* to other nodes or itself.

## 4 Layer-adaptive learning rate

DNNs are typically trained using methods such as stochastic gradient descent (SGD), which apply a fixed global learning rate (LR) across all layers. However, a fixed LR can cause inefficiencies and instabilities during training. Some adaptive update rules like AdaGrad (Duchi et al., 2011) and Adam (Kingma and Ba, 2014) adjust the global LR to mitigate these issues. However, these methods remain suboptimal for EBLL in hierarchical networks, where layer parameter updates driven by each layer's own parameter variations (Equation 4). Given the significant variation in gradient dynamics across layers in such models, a global LR fails to adequately address the distinct needs of each layer, resulting in inefficient convergence and potential training instability.

**Figure 2:**
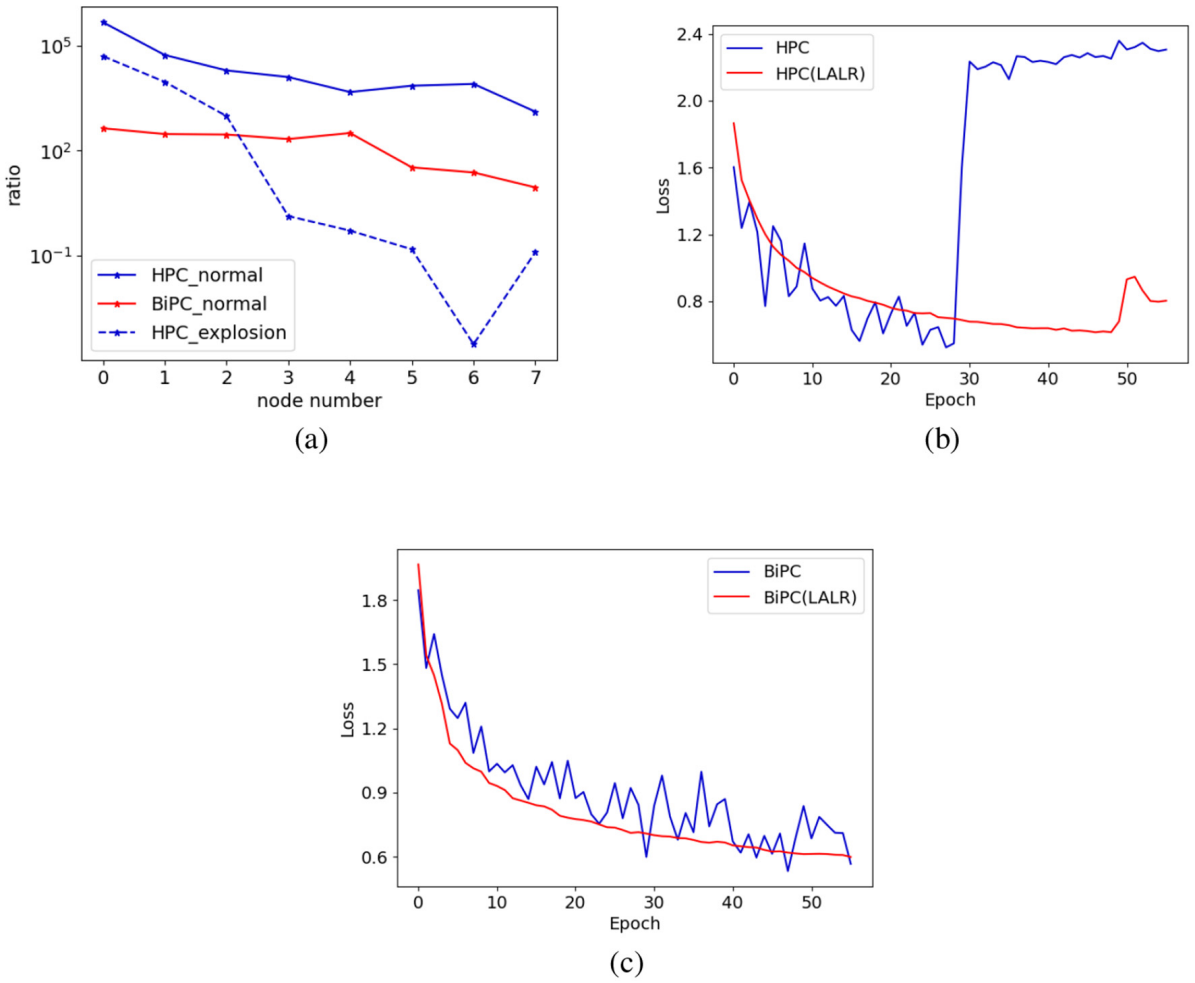
The impact of the LALR method. **(a)** The ratio of weight to gradient norms across various nodes in the model, with the y-axis showing the ratio ∥*w*∥_2_/(η∥*dw*∥_2_), and the x-axis representing different nodes. The solid lines depict the ratios for both HPC and BiPC models during the 20*th* epoch of normal training, while the dashed line indicates the ratio at the 30*th* epoch when the gradient explosion took place. The training losses of **(b)** HPC and **(c)** BiPC on the CIFAR10 dataset before and after incorporating LALR. The blue and orange lines denote Adam and LALR optimizers, respectively.

To demonstrate this point, we calculated the ratio of the model's weight norm based on the product of each node's gradient norm and the global LR. This ratio quantifies weight changes in individual nodes relative to the entire weight space. As illustrated in Figure 2a, significant variations in weight changes are observed across different nodes. As noted by (You et al. 2017), (2020), an excessively large LR can cause parameter updates across the entire parameter space to become too large, risking divergence. This observation is evident when the ratio falls below 1, a result confirmed in our experiment and depicted in Figures 2b, c.

To resolve this issue, we propose adapting the LR for each layer based on its parameter changes relative to the entire parameter space, aiming to enhance training stability. This approach forms the foundation of our Layer-Adaptive Learning Rate Optimization (LALR) algorithm. LALR introduces the layer-wise LR (η_θ, *i*_), derived from a global LR (η_θ_) and scaled to ensure stable and balanced updates across the model. The key idea is to normalize the parameter update magnitude of each layer to match the average update magnitude across the model's entire parameter space, thereby mitigating disparities in parameter change amplitudes among layers. To achieve this, we first quantify the layer-wise parameter update magnitude (Δθ_*i*_) and average update magnitude of all model parameters ($\text{Δ}\bar{\theta }$).


(13) $$\begin{array}{l} \text{Δ}\theta_{i}=\eta_{\theta ,i}\cdot ∥d\theta_{i}{∥}_{2}, \end{array}$$

(14) $$\begin{array}{l} \text{Δ}\bar{\theta }=\eta_{\theta }\cdot ∥S\sum d\theta_{i}{∥}_{2}, \end{array}$$

We employ the harmonic mean function S to measure the update magnitude across the model's parameter space. This choice is motivated by the harmonic mean's reduced sensitivity to extreme values, which ensures a more robust estimation of the gradient behavior across all model parameters. Finally, we adjust the layer-wise learning rate η_θ, *i*_ based on the relative balance between local parameter gradient updates (Δθ_*i*_) and global parameter gradient updates ($\text{Δ}\bar{\theta }$) (see Equations 15, 16).


(15) $$\begin{array}{l} \frac{\text{Δ}\theta_{i}}{\text{Δ}\bar{\theta }}=1, \end{array}$$

(16) $$\begin{array}{l} \eta_{\theta ,i}=\frac{\eta_{\theta }\cdot ∥S\sum d\theta_{i}{∥}_{2}}{∥d\theta_{i}{∥}_{2}}, \end{array}$$

The advantage of this local-global relative balance update strategy is twofold: it ensures that layers with smaller gradients receive larger layer-specific learning rates, allowing sufficient updates to local layer parameters, and that layers with larger gradients receive smaller layer-specific learning rates, preventing excessive updates in any single layer that might destabilize training. This dual benefit improves overall optimization efficiency while maintaining stability across various gradient magnitudes. As shown in Figures 2b, c, applying LALR significantly stabilizes the training process of both HPC and BiPC. However, LALR does not fully eliminate the risk of divergence in HPC, with occasional divergence emerging in the later stages of training. Nevertheless, it provides a notable improvement compared to HPC with standard SGD.

## 5 Energy-based framework

This section introduces an energy-based framework that integrates PC and equilibrium propagation (EP). EP (Scellier and Bengio, 2017), a key method in EBLL, uses an energy function combining Hopfield energy and output loss. In the first phase, EP solely minimizes the Hopfield energy, which includes the unit states of all network nodes, guiding the model to a steady state and generating a prediction. In the second phase, output loss is added to direct the model toward the correct target. Resultantly, the energy, including the output loss, is minimized again, causing a new steady state. Once this state is achieved, the model's weights are updated based on the difference between the energy gradients at the initial and second steady states.

**Figure 3:**
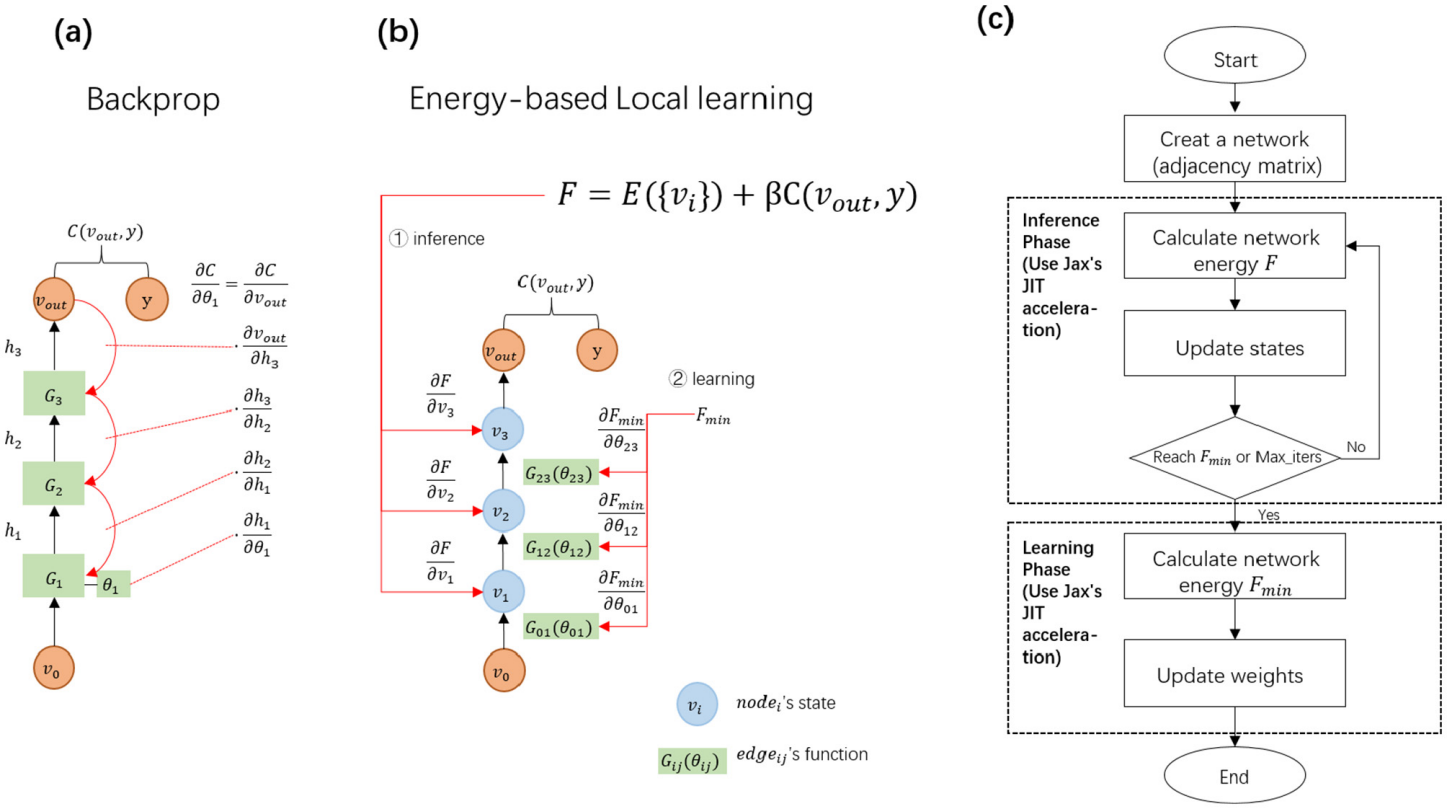
**(a)** In backprop, the error is transmitted step-by-step from the output layer to the input layer, with parameters updating based on the globally propagated errors. The green rectangle represents a layer function containing trainable parameters. **(b)** In the EBLL framework, node states are defined at the nodes, while parameters are situated on the edges. The total network energy consists of internal energy from the hidden states and external energy related to the output states. Both states and parameters can be updated locally based on the energy. **(c)** In the EBLL framework, the initial state is determined by traversing the adjacency matrix, and the network energy is computed using the energy function and the initial state. Following this process, the inference phase is performed, followed by the learning phase, to fully execute the EBLL method.

As outlined in PC and EP, the EBLL method consists of two distinct phases: first, it adjusts the model's states to minimize energy; second, it updates the network parameters once the energy reaches its minimum. This is quite different from backpropagation. Backpropagation has only one parameter adjustment stage, that is, the output layer error is adjusted by the chain rule to propagate backward layer by layer to update the learnable parameters (Figure 3a).

To effectively support EBLL, we have redefined the energy-based framework in terms of both energy form and network structure. As outlined in Section 2, any EBLL energy consists of two components:

Internal energy, which represents the energy contribution from all layers of the network except the output layer. It encapsulates the inherent dynamics of the network's internal states in the absence of influence from external supervisory signals. External energy corresponds to the supervised loss. Therefore, our framework defines the energy function as follows:


(17) $$\begin{array}{l} F=E+\beta C, \end{array}$$

where *E* and *C* indicate the internal and external energy, respectively, *E* in EP implies a kind of Hopfield energy, defined as $E=\frac{1}{2}\underset{i}{\sum }||v_{i}|{|}_{2}-\frac{1}{2}\underset{i,j,i\neq j}{\sum }\rho \left(v_{i}\right)w_{ij}\rho \left(v_{j}\right)-\underset{i}{\sum }b_{i}\rho \left(v_{i}\right)$, and *E* in PC signifies the sum of squares of the prediction error, calculated from the actual and estimated node states of the network, Here, β ∈ [0, 1] refers to a scaling factor used to balance the influence of internal energy and external energy. Specifically, When β = 0, the influence of external energy is eliminated, and the model operates in a fully free phase, stabilizing solely based on its internal dynamics. When β ∈ (0, 1), the model becomes subject to constraints from external labels at the output layer, a requirement essential for supervised tasks. The target loss function, acting as external energy, drives the model to re-establish a balance between maintaining intrinsic state stability and achieving the target objective. When β = 1, the weights of internal and external energies are equal, maximizing the external influence while preserving the integrity of the internal structure without overwhelming.

The proposed network structure and energy form enable any EBLL to operate within the framework in two stages: inference and learning stages. During the inference stage, as expressed in Equation 18, the network achieves equilibrium by adjusting *v*_*i*_ to minimize *F*. Once equilibrium is reached, the learning stage as formulated in Equation 19 optimizes synaptic weights by adjusting θ_*i*_ to further minimize *F*. Notably, this approach ensures that the network dynamics naturally align with the gradient direction of the target losses.


(18) $$\begin{array}{l} \text{inference}:dv_{i}=\frac{\partial F}{\partial v_{i}}, \end{array}$$

(19) $$\begin{array}{l} \text{learning}:d\theta_{i}=\frac{\partial F_{min}}{\partial \theta_{i}}, \end{array}$$

The network is constructed using nodes and edges as fundamental units, where nodes represent states and edges denote parameterized mapping functions. This design allows the framework to accommodate networks with arbitrary topologies, as represented in Figure 3b. An adjacency matrix is defined to record the indices of edges connecting nodes.

The EBLL framework is implemented on the Jax backend–a Python library developed by Google designed for high-performance array computation and program transformation (Frostig et al., 2018). Within this framework (Figure 3c), PC or EP sequentially performs inference and learning phases to train the model. During the inference phase, initial states and energy are computed by traversing the adjacency matrix, followed by iterative state updates until energy minimization. In the learning phase, local computations on the minimized energy enable parallel parameter updates. By utilizing JAX's Just-in-Time (JIT) technology (Bradbury et al., 2018), operations such as automatic differentiation of any order–including those expressed in Equations 18, 19 are efficiently compiled. This process converts numerical computations in the prediction process into an optimized machine code at runtime using advanced tracing and XLA compilers, as demonstrated in Appendices A and B.

## 6 Experiment

Our methods are trained and tested for object recognition using specific datasets and networks, with performance compared against baselines. All experiments are conducted within our energy-based framework.

### 6.1 Experiment settings

#### 6.1.1 Datasets

##### 6.1.1.1 MNIST

This dataset comprises 70,000 grayscale images of handwritten digits, each measuring 28*28 pixels and representing single digits ranging from 0 to 9. The dataset is divided into training 60,000 images and 10,000 testing images. Preprocessing involves normalizing all images using channel means and standard deviations.

##### 6.1.1.2 CIFAR

This dataset includes two main subsets: CIFAR10 and CIFAR100, containing 32*32 colored images drawn from 10 and 100 classes, respectively. Each subset comprises 50,000 training images and 10,000 testing images. Preprocessing involves data normalization and augmentation techniques such as flipping and random cropping.

##### 6.1.1.3 Tiny ImageNet

A curated subset of the larger ImageNet dataset, Tiny ImageNet consists of 100,000 color images at a resolution of 64*64 pixels. The dataset features 200 distinct classes, each comprising 500 training images, 50 validation images, and 50 test images.

#### 6.1.2 Network architecture

We use network architectures with different spatial and temporal complexity: hierarchical feedforward network and skip connection recurrent network, as represented in Table 1.

**Table 1:** Network configuration.

**Architecture**	**Hierarchical feedforward network**	**Skip connection recurrent network**	
		**Simple**	**Complex**
Number of nodes	11	8	8
Time	–	5	5
Nodes' connections	conv3-128	conv3-64^**^	conv3-32^**^
	conv3-256 maxpool-2	conv3-128^**^	conv3-64^**^
	conv3-512 maxpool-2	conv3-128^*^ conv3-256^**^ conv3-512^***^	conv1-128# maxpool-2# conv3-128^*^ conv3-256^**^ conv3-1024^*^^**^
	conv3-120 maxpool-2	conv3-256^*^ conv3-512^**^ conv3-200^***^	conv1-512# conv3-512^*^ conv3-1024^**^ conv3-256^***^
	conv1-256	conv3-512^*^ conv3-200^**^ conv3-32^*^^**^	conv1-768# maxpool-2# conv3-768^*^ conv3-256^**^ conv3-256^*^^**^
	conv1-80	conv3-200^*^ conv3-32^**^	conv1-128# conv3-128^*^ conv3-256^**^
	conv3-64	conv3-32^*^	conv1-64# maxpool-2# conv3-64^*^
	conv3-100	flatten fc-10/100/200	flatten fc-512 fc-10/100/200
	conv3-50 maxpool-2		
	fc-512		
	fc-10/100/200		

^*^Recurrent connection; ^**^forward connection; ^***^skip connection; ^#^internal connection.

##### 6.1.2.1 Hierarchical feedforward network

his network architecture is a feedforward convolutional neural network architecture comprising nine convolutional layers and two fully connected layers. The convolutional layers utilize 3*3 and 1*1 kernels with varying kernel counts per layer. Max pooling with a 2*2 kernel the size of the feature maps, followed by the application of the tanh activation function. Two fully connected layers follow the convolutional layers.

##### 6.1.2.2 Skip connection recurrent network

As shown in Figure 1b, the network has two variants based on spatial complexity: a simple version and a complex version. The simple version consists of 8 nodes, with the 0*th* node serving as the input. Each node has a single state, and the 0*th* node encodes the input to the 1*th* node using a convolution function, mimicking the retina's processing of visual input. The 1*th* node processes the input, repeating the signal and projecting it to the second high-level node via the convolution function. The 7*th* node decodes the output. Apart from the 1*th* and 7*th* nodes, each internal node has edge functions that map the state to the next high-level, time step, and cross-layer nodes, using 3*3 convolutions with varying channels. The complex version also uses eight nodes, with each internal node containing two states. Compared to the simple version, the edge functions include an additional internal state mapping function, implemented as a 1*1 convolution function. These convolution settings are inspired by CORnet (Kubilius et al., 2018) settings. Additionally, the nonlinear activation function employs the hyperbolic tangent (tanh) activation function in each layer.

#### 6.1.3 Hyper-parameter

The model was trained and tested using an NVIDIA A100 80G GPU device, with the remaining hyperparameters detailed in Table 2.

**Table 2:** Settings for model hyperparameters.

**Parameter**	**Description**	**Value**
*Batchsize*	Number of samples per gradient update	128
*N*	Maximum number of iterations for inference phase	200
η_*v*_	LR for inference phase	0.01
η_θ_	LR for learning phase	0.01
β	Scaling factor for external energy	1
*Threshold*	Energy convergence threshold	1e–7

### 6.2 Evaluation of the effectiveness of BiPC

In the EBLL model, the inference and learning phases are executed sequentially. During inference, gradient descent is applied to the energy function to minimize energy by adjusting the states. Once minimized, the weight gradient is computed to update the parameters. Managing gradient explosion or vanishing during inference is critical, as these issues indicate extreme energy values and directly affect weight updates in the learning phase. Proper gradient control during inference ensures stable and effective parameter optimization.

**Figure 4:**
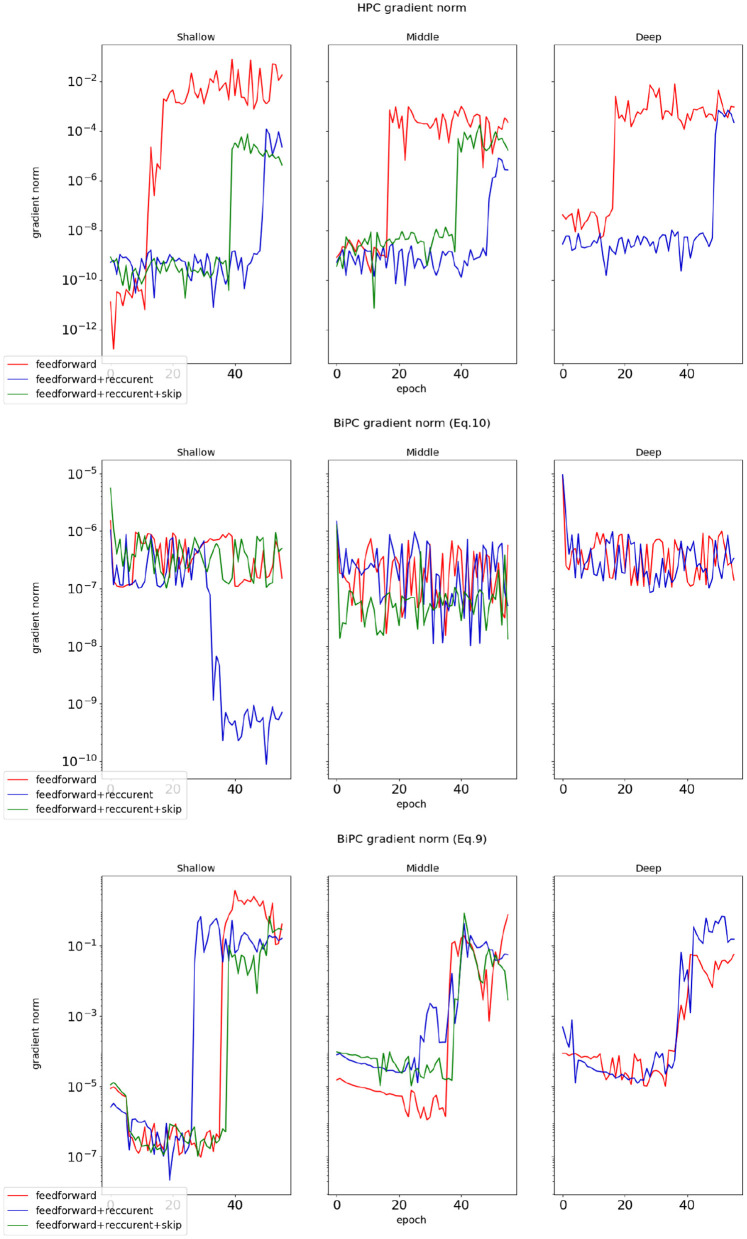
The gradient norms of HPC and BiPC were evaluated across varying network depths and connection structures during the inference phase. The average gradient norm was determined for the 2*th* (shallow), 4*th* (middle), and 6*th* (deep) nodes, with the 6*th* node not incorporating skip connections. The **Top subplot**: the gradient norms for the HPC model. The **Middle subplot**: the BiPC gradients derived from the energy formulation expressed in Equation 10. The **Bottom subplot**: the gradient norms of the BiPC model based on the energy formulation presented in Equation 9.

**Figure 5:**
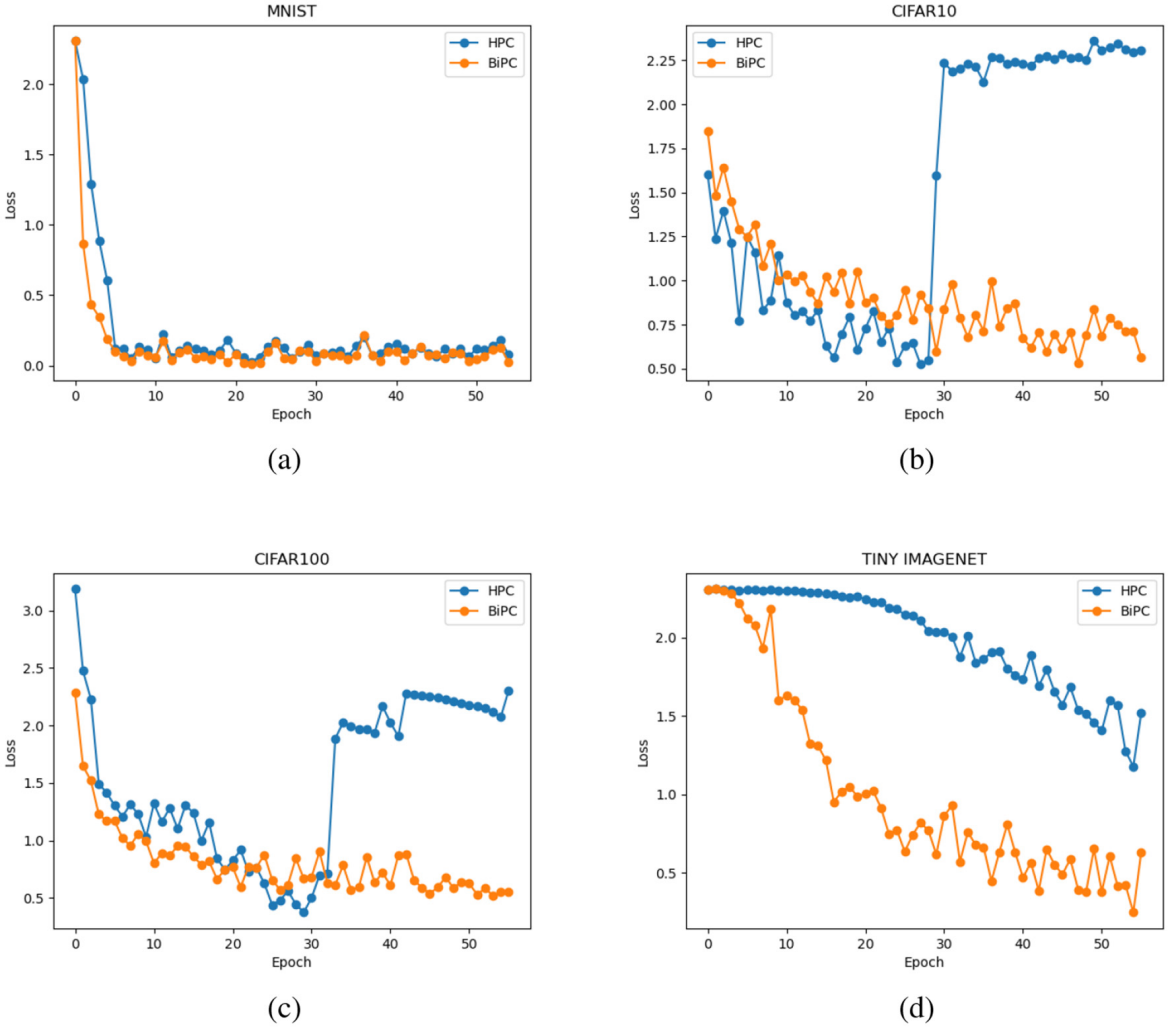
Training loss comparison between the HPC and BiPC models across four benchmark datasets: **(a)** MNIST, **(b)** CIFAR10, **(c)** CIFAR100, and **(d)** Tiny ImageNet. Both HPC and BiPC are trained using the simple skip connection recurrent network (see Table 1) with the Adam optimizer. In all subfigures, the blue curve represents the HPC model, while the orange curve represents the BiPC model.

We examine whether the BiPC model, utilizing two distinct energy formulas expressed in Equations 9, 10, can effectively mitigate gradient explosion and vanishing during inference. The gradient norm value during training serves as the primary indicator for detecting these issues. A near-zero gradient norm indicates vanishing gradients, while a sudden escalation by several orders of magnitude signals gradient explosion. Using the simple version of the skip connection recurrent network from Table 1 and the CIFAR10 dataset as an example, we evaluate BiPC under varying network depths and connection configurations. The average gradient norm during the inference phase is computed. As illustrated in Figure 4, the red line represents the simple version of the skip connection recurrent network without recurrent or skip connections, while the blue line depicts the same network without skip connections. The green line corresponds to the fully equipped skip connection recurrent network (simple version). To evaluate HPC's performance with gradients across varying depths and connection types, the top subplot of Figure 4 illustrates that HPC consistently experiences gradient explosion, regardless of whether connections are feedforward, combined feedforward and recurrent, or include skip connections. The middle subplot of Figure 4 demonstrates that with the energy equation in Equation 10, gradient explosion is effectively mitigated across various depths and connections. However, gradient vanishing remains an issue in shallower layers, especially without skip connections. This trend indicates that while the BiPC model resolves gradient explosion, addressing vanishing gradients requires the inclusion of skip connections. The bottom subfigure of Figure 4 reveals that the BiPC model using Equation 9 experiences the gradient explosion across various depths and connections. Similarly, we evaluate HPC and BiPC learning performance on MNIST, CIFAR10, CIFAR100, and Tiny ImageNet using the simple skip connection recurrent network and the Adam optimizer. As illustrated in Figure 5, the BiPC model demonstrates effectiveness by mitigating the loss explosion on CIFAR10/100 and surpassing HPC performance on more complex datasets such as Tiny ImageNet.

### 6.3 Evaluation of the effectiveness of LALR

**Figure 6:**
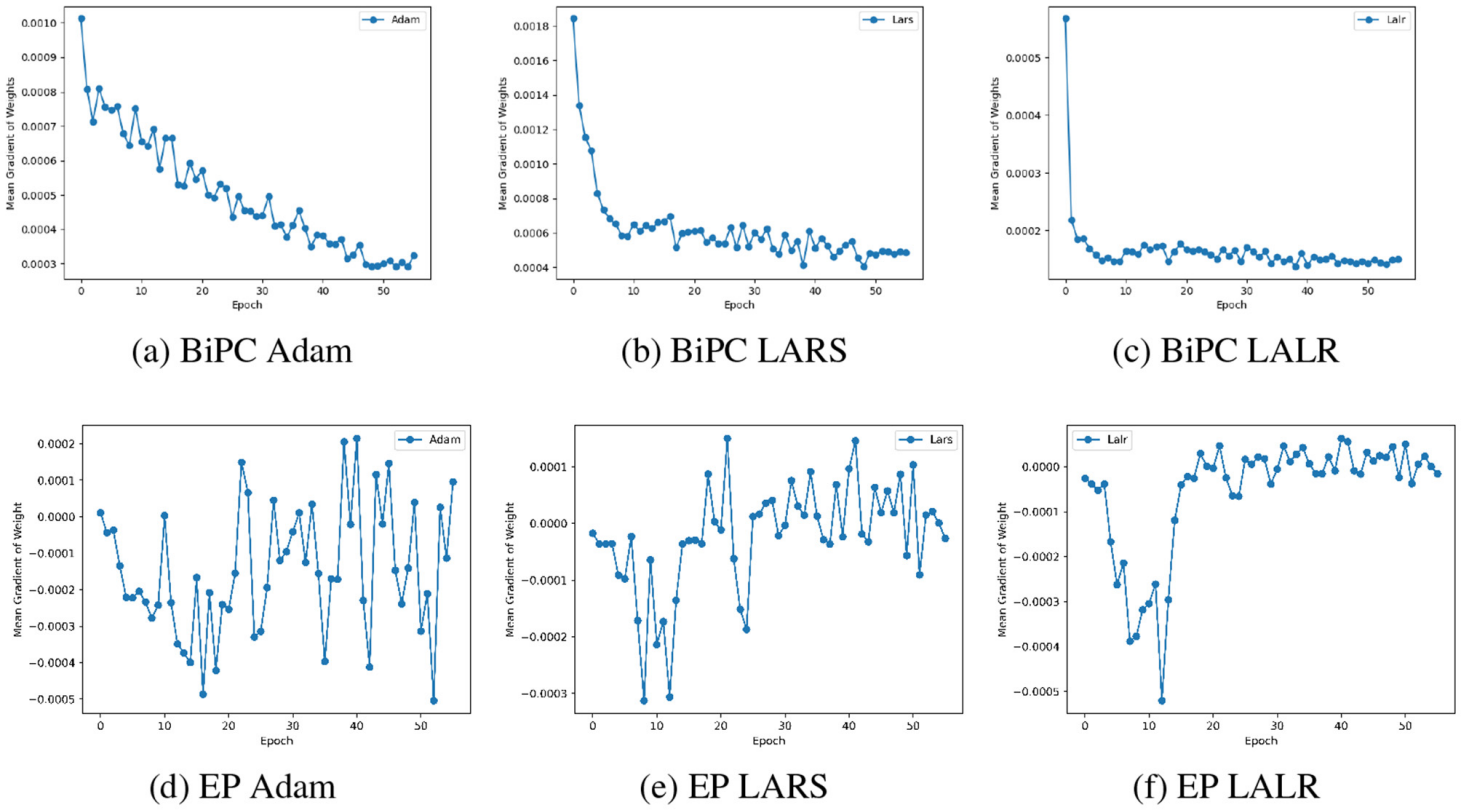
Comparison of weight gradient fluctuations across various optimizers on the CIFAR10 dataset. Changes in weight gradients for the BiPC model when utilizing **(a)** Adam, **(b)** LARS, and **(c)** LALR optimizers. Similar plots for EP corresponding to a **(d)** EP Adam, **(e)** EP LARS, and **(f)** EP LALR optimizers.

To evaluate the effectiveness of the LALR method, we analyzed weight gradient fluctuations and accuracy for BiPC/EP across three adaptive optimization algorithms: Adam, LALR, and LARS (You et al., 2017). Figures 6a–c demonstrate the variations in weight gradients for BiPC when employing these optimizers within the 4*th* block of the generalized skip connection recurrent architecture. Figures 6d–f depict the weight gradient variations for EP with different optimizers in the first layer of the hierarchical feedforward network. LALR ensures smoother, more stable gradient transitions and achieves stability more rapidly than LARS. Table 3 presents the Top-1 validation accuracies of BiPC and LALR, respectively, compared against HPC, EP and backprop baselines across various datasets. The results indicate that LALR substantially enhances the accuracy of HPC, BiPC, and EP. Notably, BiPC combined with LALR achieves accuracy levels comparable to backprop within the same network architecture. However, we also observe lower performance on CIFAR-100 and Tiny ImageNet compared to MNIST and CIFAR-10. This discrepancy can be attributed to several factors. First, CIFAR-100 and Tiny ImageNet contain 100 and 200 categories respectively, with significant intra-class variability in object appearance, posture, and background. In contrast, MNIST and CIFAR-10 have only 10 well-separated categories with simpler visual patterns. The higher data complexity in CIFAR-100 and Tiny ImageNet increases the learning difficulty under fixed model capacity. Second, complex datasets often require deeper or wider networks with stronger feature representation capabilities to capture fine-grained distinctions between classes. As shown in Table 3, the skip-connection recurrent architecture, which contains more parameters and a more expressive structure than the hierarchical feedforward model, consistently outperforms the latter across all datasets. Third, our model inherits the Gaussian distribution assumption from the free-energy-based HPC framework. While this simplifies energy formulation and allows tractable optimization, it limits expressiveness when applied to high-resolution or highly non-Gaussian data. In real-world datasets such as CIFAR-100 and Tiny ImageNet, the pixel distributions are multimodal and deviate significantly from Gaussianity, which may lead to increased variational error and degraded performance.

**Table 3:** Comparison of validation accuracies (%) for various approaches on the hierarchical feedforward and the skip connection recurrent networks.

**Network**	**Methods**	**MNIST**	**CIFAR10**	**CIFAR100**	**Tiny ImageNet**
Hierarchical feedforward network	HPC (Adam)	96.91 ± 0.30	60.96 ± 6.53	33.59 ± 4.30	21.06 ± 5.25
	BiPC (Adam)	98.46 ± 0.00	80.39 ± 0.70	49.51 ± 0.89	35.98 ± 0.63
	EP (Adam)	96.42 ± 0.40	75.17 ± 0.73	44.14 ± 0.65	30.49 ± 0.89
	backprop (Adam)	97.63 ± 0.00	81.69 ± 0.01	52.47 ± 0.01	35.70 ± 0.00
	HPC (LALR)	97.98 ± 0.02	70.61 ± 2.25	36.20 ± 2.40	24.82 ± 1.96
	BiPC (LALR)	98.82 ± 0.00	83.95 ± 0.36	53.12 ± 0.55	37.85 ± 0.49
	EP (LALR)	98.60 ± 0.06	81.56 ± 0.54	54.52 ± 0.56	35.22 ± 0.60
	backprop (LALR)	98.95 ± 0.00	82.91 ± 0.00	54.01 ± 0.02	36.14 ± 0.01
Skip connection recurrent network (simple)	HPC (Adam)	95.63 ± 0.36	62.21 ± 5.13	33.94 ± 5.62	23.05 ± 4.30
	BiPC (Adam)	95.03 ± 0.26	87.30 ± 0.75	76.58 ± 0.60	67.06 ± 0.63
	backprop (Adam)	98.84 ± 0.05	90.05 ± 0.07	77.63 ± 0.10	70.83 ± 0.26
	HPC (LALR)	97.66 ± 0.01	64.02 ± 3.53	34.98 ± 3.50	25.16 ± 3.69
	BiPC (LALR)	99.22 ± 0.01	90.82 ± 0.48	81.70 ± 0.47	72.39 ± 0.53
	backprop (LALR)	98.89 ± 0.02	91.46 ± 0.42	82.40 ± 0.55	71.98 ± 0.55
Skip connection recurrent network (complex)	HPC (Adam)	96.03 ± 0.03	63.33 ± 5.03	34.20 ± 4.68	24.82 ± 4.93
	BiPC (Adam)	97.43 ± 0.00	90.63 ± 0.42	79.87 ± 0.47	70.25 ± 0.50
	backprop (Adam)	98.51 ± 0.00	94.25 ± 0.43	80.65 ± 0.45	70.58 ± 0.49
	HPC (LALR)	98.65 ± 0.01	66.13 ± 2.99	35.80 ± 3.49	26.61 ± 4.20
	BiPC (LALR)	99.22 ± 0.00	93.78 ± 0.40	83.96 ± 0.46	73.35 ± 0.45
	backprop (LALR)	98.89 ± 0.01	94.56 ± 0.46	81.60 ± 0.49	74.18 ± 0.50

Bold values indicate the best accuracy results.

### 6.4 Evaluation of the effectiveness of energy-based framework

**Figure 7:**
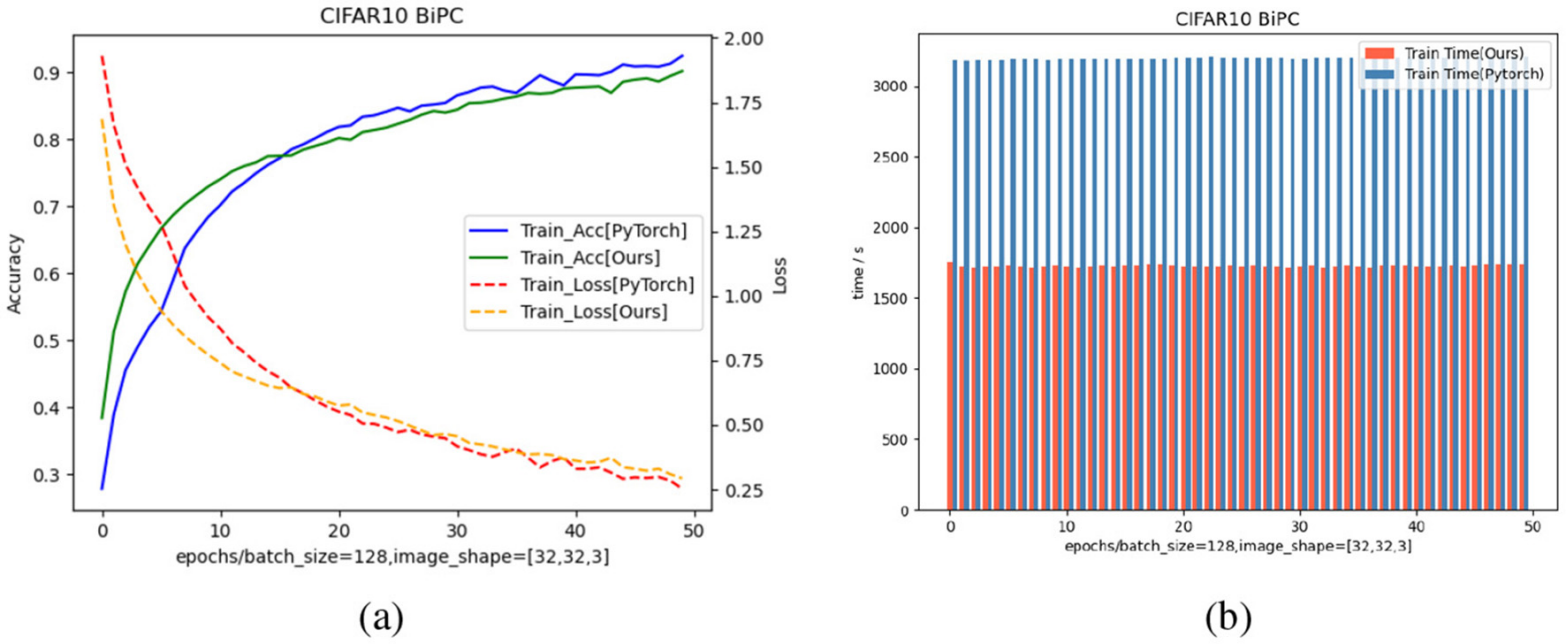
Comparison of training accuracy, loss, and runtime between the our energy-based model with JAX implementation and the same model with PyTorch implementation on the CIFAR10 dataset. **(a)** Training curves of accuracy and loss using a batch size of 128 and input shape [32,32,3]. Solid curves denote training accuracy, while dashed curves denote training loss. Blue solid curve and red dashed curve correspond to our BiPC model with standard PyTorch implementation, whereas the green solid curve and orange dashed curve correspond to our BiPC model with JAX implementation. **(b)** Runtime comparison per epoch. The red bars indicate the training time of our BiPC model with JAX implementation, while the blue bars show the PyTorch baseline. Across all epochs, our method significantly reduces computational time, demonstrating better efficiency.

To assess the reliability and efficiency of our framework, we trained the specified network using identical methods and hyperparameters on both PyTorch and our framework, utilizing a single NVIDIA A100 GPU. As shown in Figure 7, our framework achieves comparable training accuracy and loss to PyTorch under identical settings, while reducing runtime by 50 percent.

## 7 Discussion and conclusion

This study aims to address gradient explosion and vanishing issues in EBLL models, such as classic hierarchical PC, during ANN training. To enhance training efficiency, we developed a JAX-based framework. Drawing on neuroscience and AI engineering, we introduce a novel BiPC model based on a biologically inspired ANN. BiPC utilizes energy from forward and backward processes to constrain updates and prevent gradient explosion during local state and parameter optimization. Experiments reveal that while bidirectional energy constraints effectively address gradient explosion in deep ANNs, resolving gradient vanishing necessitates incorporating skip connections. BiPC with recurrent and skip connections surpasses models relying solely on feedforward and feedback connections. To address gradient variability across layers during local updates, we propose the LALR method, optimizing gradient descent for state and parameter updates in energy-based PC and EP. This method significantly improves target recognition performance. BiPC with LALR achieves object recognition accuracy comparable to backprop; however, LALR alone cannot fully address gradient explosion in hierarchical PC. Our EBLL framework, structured with points and edges, demonstrates superior accuracy and faster execution compared to PyTorch in hierarchical and recurrent architectures.

Our model and framework have some limitations. While BiPC resolves gradient explosion, it does not fully address gradient vanishing, likely due to both model directions producing low energy, leading to gradient decay. Additionally, BiPC focuses on a single cortical region without accounting for inter-unit interactions across the entire brain. Furthermore, this model is trained solely on biologically inspired neural networks, excluding biomimetic networks such as spiking neural networks (SNNs) or brain emulation networks. This study focuses on optimizing EBLL algorithms for practical applications in ANNs, rather than theoretical study. Future work will investigate energy-based models in biomimetic networks, such as SNNs and brain emulation systems. Additionally, the bidirectional concept in the BiPC method has yet to be effectively applied to local learning approaches based on Hopfield energy, such as EP. This trend is because Hopfield energy originates from a fully connected network, making its energy undirected. Additionally, the EBLL framework uses a graph structure, theoretically enabling its application to networks with arbitrary topologies, including large-scale brain simulation networks with millions of nodes and edges. However, the framework lacks effective memory management, limiting its scalability for large networks. Improving efficient learning support for large-scale networks is a critical challenge. Currently, brain simulation networks (Potjans and Diesmann, 2014) have not successfully handled complex tasks such as vision and text. Therefore, overcoming these limitations and advancing EBLL methods for intelligent task mastery in large-scale brain simulations is a key research objective.

## Data Availability

The original contributions presented in the study are included in the article/Supplementary material, further inquiries can be directed to the corresponding author.

## References

[B1] Amit Y. (2019). Deep learning with asymmetric connections and hebbian updates. Front. Comput. Neurosci. 13:18. 10.3389/fncom.2019.00018 31019458 PMC6458299

[B2] Angelucci A. Petreanu L. (2023). “Feedforward and feedback connections: functional connectivity, synaptic physiology, and function,” in The Cerebral Cortex and Thalamus, 405–418. 10.1093/med/9780197676158.003.0038

[B3] Bengio Y. (2014). How auto-encoders could provide credit assignment in deep networks via target propagation. arXiv [Preprint]. arXiv:1407.7906. 10.48550/arXiv.1407.7906

[B4] Bengio Y. Fischer A. (2015). Early inference in energy-based models approximates back-propagation. arXiv [Preprint]. arXiv:1510.02777. 10.48550/arXiv.1510.02777

[B5] Boccato T. Ferrante M. Duggento A. Toschi N. (2024). Beyond multilayer perceptrons: Investigating complex topologies in neural networks. Neural Netw. 171, 215–228. 10.1016/j.neunet.2023.12.012 38096650

[B6] Bradbury J. Frostig R. Hawkins P. Johnson M. J. Leary C. Maclaurin D.. (2018). JAX: Composable Transformations of Python+*NumPy Programs*. Available online at: http://github.com/google/jax

[B7] Buckley C. L. Kim C. S. McGregor S. Seth A. K. (2017). The free energy principle for action and perception: a mathematical review. J. Math. Psychol. 81, 55–79. 10.1016/j.jmp.2017.09.004

[B8] Cheng A. Ping H. Wang Z. Xiao X. Yin C. Nazarian S.. (2024). “Unlocking deep learning: a bp-free approach for parallel block-wise training of neural networks,” in ICASSP 2024-2024 IEEE International Conference on Acoustics, Speech and Signal Processing (ICASSP) (Seoul: IEEE), 4235–4239. 10.1109/ICASSP48485.2024.10447377

[B9] Clark A. (2013). Whatever next? Predictive brains, situated agents, and the future of cognitive science. Behav. Brain Sci. 36, 181–204. 10.1017/S0140525X12000477 23663408

[B10] Crick F. (1989). The recent excitement about neural networks. Nature 337, 129–132. 10.1038/337129a0 2911347

[B11] Dold D. Kungl A. F. Sacramento J. Petrovici M. A. Schindler K. Binas J.. (2019). Lagrangian dynamics of dendritic microcircuits enables real-time backpropagation of errors. Target 100:2.

[B12] Duchi J. Hazan E. Singer Y. (2011). Adaptive subgradient methods for online learning and stochastic optimization. J. Mach. Learn. Res. 12, 2121–2159. 10.5555/1953048.2021068

[B13] Friston K. (2003). Learning and inference in the brain. Neural Netw. 16, 1325–1352. 10.1016/j.neunet.2003.06.005 14622888

[B14] Friston K. (2005). A theory of cortical responses. Philos. Trans. R. Soc. B: Biol. Sci. 360, 815–836. 10.1098/rstb.2005.1622 15937014 PMC1569488

[B15] Friston K. (2010). The free-energy principle: a unified brain theory? Nat. Rev. Neurosci. 11, 127–138. 10.1038/nrn2787 20068583

[B16] Friston K. Kiebel S. (2009). Predictive coding under the free-energy principle. Philos. Trans. R. Soc. B: Biol. Sci. 364, 1211–1221. 10.1098/rstb.2008.0300 19528002 PMC2666703

[B17] Friston K. Kilner J. Harrison L. (2006). A free energy principle for the brain. J. Physiol. 100, 70–87. 10.1016/j.jphysparis.2006.10.001 17097864

[B18] Friston K. J. Stephan K. E. (2007). Free-energy and the brain. Synthese 159, 417–458. 10.1007/s11229-007-9237-y 19325932 PMC2660582

[B19] Frostig R. Johnson M. J. Leary C. (2018). Compiling machine learning programs via high-level tracing. Syst. Mach. Learn. 4, 1–3.

[B20] Hinton G. (2003). The ups and downs of hebb synapses. Can. Psychol./Psychol. Canad. 44:10. 10.1037/h0085812

[B21] Hinton G. (2022). The forward-forward algorithm: some preliminary investigations. arXiv [Preprint]. arXiv:2212.13345. 10.48550/arXiv.2212.13345

[B22] Hirani G. Kevin I. Wang K. Abdulla W. (2024). A scalable unsupervised and back propagation free learning with sacsom: a novel approach to SOM-based architectures. IEEE Trans. Artif. Intell. 6, 955–967. 10.1109/TAI.2024.3504479

[B23] Hoffmann C. Cho E. Zalesky A. Di Biase M. A. (2024). From pixels to connections: exploring *in vitro* neuron reconstruction software for network graph generation. Commun. Biol. 7:571. 10.1038/s42003-024-06264-9 38750282 PMC11096190

[B24] Hopfield J. J. (1982). Neural networks and physical systems with emergent collective computational abilities. Proc. Nat. Acad. Sci. 79, 2554–2558. 10.1073/pnas.79.8.2554 6953413 PMC346238

[B25] Khacef L. Miramond B. Barrientos D. Upegui A. (2019). “Self-organizing neurons: toward brain-inspired unsupervised learning,” in 2019 International Joint Conference on Neural Networks (IJCNN) (Budapest: IEEE), 1–9. 10.1109/IJCNN.2019.8852098

[B26] Kinghorn P. F. Millidge B. Buckley C. L. (2022). “Preventing deterioration of classification accuracy in predictive coding networks,” in International Workshop on Active Inference (Cham: Springer), 1–15. 10.1007/978-3-031-28719-0_1

[B27] Kingma D. P. Ba J. (2014). Adam: a method for stochastic optimization. arXiv [Preprint]. arXiv:1412.6980. 10.48550/arXiv.1412.6980

[B28] Krotov D. Hopfield J. J. (2019). Unsupervised learning by competing hidden units. Proc. Nat. Acad. Sci., 116, 7723–7731. 10.1073/pnas.1820458116 30926658 PMC6475390

[B29] Kubilius J. Schrimpf M. Nayebi A. Bear D. Yamins D. L. DiCarlo J. J.. (2018). Cornet: modeling the neural mechanisms of core object recognition. bioRxiv. 10.1101/408385

[B30] Lee D.-H. Zhang S. Fischer A. Bengio Y. (2015). “Difference target propagation,” in Machine Learning and Knowledge Discovery in Databases: European Conference, ECML PKDD 2015, Porto, Portugal, September 7-11, 2015, Proceedings, Part I 15 (Cham: Springer), 498–515. 10.1007/978-3-319-23528-8_31

[B31] Lillicrap T. P. Cownden D. Tweed D. B. Akerman C. J. (2016). Random synaptic feedback weights support error backpropagation for deep learning. Nat. Commun. 7:13276. 10.1038/ncomms13276 27824044 PMC5105169

[B32] Lillicrap T. P. Santoro A. Marris L. Akerman C. J. Hinton G. (2020). Backpropagation and the brain. Nat. Rev. Neurosci. 21, 335–346. 10.1038/s41583-020-0277-3 32303713

[B33] Luo H. Fu J. Glass J. (2017). Adaptive bidirectional backpropagation: towards biologically plausible error signal transmission in neural networks. arXiv [Preprint]. arXiv:1702.07097. 10.48550/arXiv.1702.07097

[B34] Millidge B. Seth A. Buckley C. L. (2021). Predictive coding: a theoretical and experimental review. arXiv [Preprint]. arXiv:2107.12979. 10.48550/arXiv.2107.12979

[B35] Millidge B. Song Y. Salvatori T. Lukasiewicz T. Bogacz R. (2023). “Backpropagation at the infinitesimal inference limit of energy-based models: unifying predictive coding, equilibrium propagation, and contrastive Hebbian learning,” in The Eleventh International Conference on Learning Representations (Kigali). Available online at: https://openreview.net/forum?id=nIMifqu2EO

[B36] Millidge B. Tschantz A. Buckley C. L. (2022). Predictive coding approximates backprop along arbitrary computation graphs. Neural Comput. 34, 1329–1368. 10.1162/neco_a_01497 35534010

[B37] Momeni A. Rahmani B. Malléjac, M. Del Hougne P. Fleury R. (2023). Backpropagation-free training of deep physical neural networks. Science 382, 1297–1303. 10.1126/science.adi8474 37995209

[B38] Moraitis T. Toichkin D. Journé, A. Chua Y. Guo Q. (2022). Softhebb: Bayesian inference in unsupervised hebbian soft winner-take-all networks. Neuromorphic Comput. Eng. 2:044017. 10.1088/2634-4386/aca710

[B39] Nøkland A. (2016). “Direct feedback alignment provides learning in deep neural networks,” in Advances in Neural Information Processing Systems, eds. D. Lee, M. Sugiyama, U. Luxburg, I. Guyon, and R. Garnett (Barcelona: Curran Associates, Inc.), Available online at: https://proceedings.neurips.cc/paper_files/paper/2016/file/d490d7b4576290fa60eb31b5fc917ad1-Paper.pdf

[B40] Piekarski M. (2023). Incorporating (variational) free energy models into mechanisms: the case of predictive processing under the free energy principle. Synthese 202:58. 10.1007/s11229-023-04292-2

[B41] Pinchetti L. Salvatori T. Yordanov Y. Millidge B. Song Y. Lukasiewicz T. (2022). “Predictive coding beyond Gaussian distributions” in Advances in Neural Information Processing Systems, eds. A. H. Oh, A. Agarwal, D. Belgrave, and K. Cho (New Orleans, LA). Available online at: https://openreview.net/forum?id=Ryy7tVvBUk

[B42] Pogodin R. Latham P. (2020). Kernelized information bottleneck leads to biologically plausible 3-factor hebbian learning in deep networks. Adv. Neural Inf. Process. Syst. 33, 7296–7307.

[B43] Potjans T. C. Diesmann M. (2014). The cell-type specific cortical microcircuit: relating structure and activity in a full-scale spiking network model. Cereb. Cortex 24, 785–806. 10.1093/cercor/bhs358 23203991 PMC3920768

[B44] Rao R. P. Ballard D. H. (1999). Predictive coding in the visual cortex: a functional interpretation of some extra-classical receptive-field effects. Nat. Neurosci. 2, 79–87. 10.1038/4580 10195184

[B45] Rockland K. S. (2022). Notes on visual cortical feedback and feedforward connections. Front. Syst. Neurosci. 16:784310. 10.3389/fnsys.2022.784310 35153685 PMC8831541

[B46] Rosenbaum R. (2022). On the relationship between predictive coding and backpropagation. PLoS ONE 17:e0266102. 10.1371/journal.pone.0266102 35358258 PMC8970408

[B47] Sa-Couto L. Wichert A. (2023). Self-organizing maps on “what-where” codes towards fully unsupervised classification. Biol. Cybern. 117, 211–220. 10.1007/s00422-023-00963-y 37188974 PMC10258173

[B48] Salova A. Kovács I. A. (2025). Combined topological and spatial constraints are required to capture the structure of neural connectomes. Netw. Neurosci. 9, 181–206. 10.1162/netn_a_00428 40161988 PMC11949549

[B49] Scellier B. Bengio Y. (2017). Equilibrium propagation: bridging the gap between energy-based models and backpropagation. Front. Comput. Neurosci. 11:24. 10.3389/fncom.2017.00024 28522969 PMC5415673

[B50] Spratling M. W. (2017). A review of predictive coding algorithms. Brain Cogn. 112, 92–97. 10.1016/j.bandc.2015.11.003 26809759

[B51] Stork D. G. (1989). “Is backpropagation biologically plausible,” in International Joint Conference on Neural Networks, Volume 2 (Washington, DC: IEEE), 241–246. 10.1109/IJCNN.1989.118705

[B52] Whittington J. C. Bogacz R. (2017). An approximation of the error backpropagation algorithm in a predictive coding network with local hebbian synaptic plasticity. Neural Comput. 29, 1229–1262. 10.1162/NECO_a_00949 28333583 PMC5467749

[B53] Xiao X. Chen H. Bogdan P. (2021). Deciphering the generating rules and functionalities of complex networks. Sci. Rep. 11:22964. 10.1038/s41598-021-02203-4 34824290 PMC8616909

[B54] Xie X. Seung H. S. (2003). Equivalence of backpropagation and contrastive hebbian learning in a layered network. Neural Comput. 15, 441–454. 10.1162/089976603762552988 12590814

[B55] Yang R. Sala F. Bogdan P. (2021). Hidden network generating rules from partially observed complex networks. Commun. Phys. 4:199. 10.1038/s42005-021-00701-5

[B56] Yin C. Cheng M. Xiao X. Chen X. Nazarian S. Irimia A.. (2023). Leader-follower neural networks with local error signals inspired by complex collectives. arXiv [Preprint] arXiv:2310.07885. 10.48550/arXiv.2310.07885

[B57] Yin C. Xiao X. Balaban V. Kandel M. E. Lee Y. J. Popescu G.. (2020). Network science characteristics of brain-derived neuronal cultures deciphered from quantitative phase imaging data. Sci. Rep. 10:15078. 10.1038/s41598-020-72013-7 32934305 PMC7492189

[B58] You Y. Gitman I. Ginsburg B. (2017). Large batch training of convolutional networks. arXiv [Preprint] arXiv:1708.03888. 10.48550/arXiv.1708.03888

[B59] You Y. Li J. Reddi S. Hseu J. Kumar S. Bhojanapalli S.. (2020). “Large batch optimization for deep learning: training BERT in 76 minutes,” in International Conference on Learning Representations (Addis Ababa). Available online at: https://openreview.net/forum?id=Syx4wnEtvH

[B60] Znaidi M. R. Sia J. Ronquist S. Rajapakse I. Jonckheere E. Bogdan P.. (2023). A unified approach of detecting phase transition in time-varying complex networks. Sci. Rep. 13:17948. 10.1038/s41598-023-44791-3 37864007 PMC10589276

