# Supplementary material for: Effective methods and framework for energy-based local learning of deep neural networks
Source: Front Artif Intell. 2025 Aug 26;8:1605706. doi: 10.3389/frai.2025.1605706 (PMC12418518; doi:10.3389/frai.2025.1605706)
Supplement: Supplementary file 1 [file Supplementary_file_1.pdf]

Supplementary Material

1 THE JAX BACKEND'S ENERGY-BASED FRAMEWORK FOR CONSTRUCTING NETWORKS

To unify programming styles and streamline the use of the energy-based framework, this framework inherits a Module class, akin to PyTorch's `nn.Module`, for network construction. As depicted in Fig. S1, the base `Module` class, located on the far left, serves as the foundation for all neural network modules, followed by sub-modules defining edge operators. Additionally, custom classes such as `RModule`, derived from `Module`, introduce bidirectional operations specific to energy models while maintaining the sequential propagation of network sub-modules. These pre-configured classes eliminate the need for manual implementation of backward operations. These classes automatically reverse the forward computations to perform the backward calculations. By inheriting from the `RModule` class, users can effortlessly transform a network with trainable parameters into a local, bidirectional energy model. `RLinear` and `RConv` define bidirectional linear and convolution operators, respectively, with their backward operations corresponding to linear and transposed convolution, respectively. Extending the `RModule` class allows for constructing networks, such as a simplified bidirectional fully connected network. This approach maintains a PyTorch-like programming style, with networks defined by edges and nodes. The edge operator (`RLinear`) in the sub-module defines the network, with an adjacency matrix attribute specifying the source and target nodes for feedforward operations.

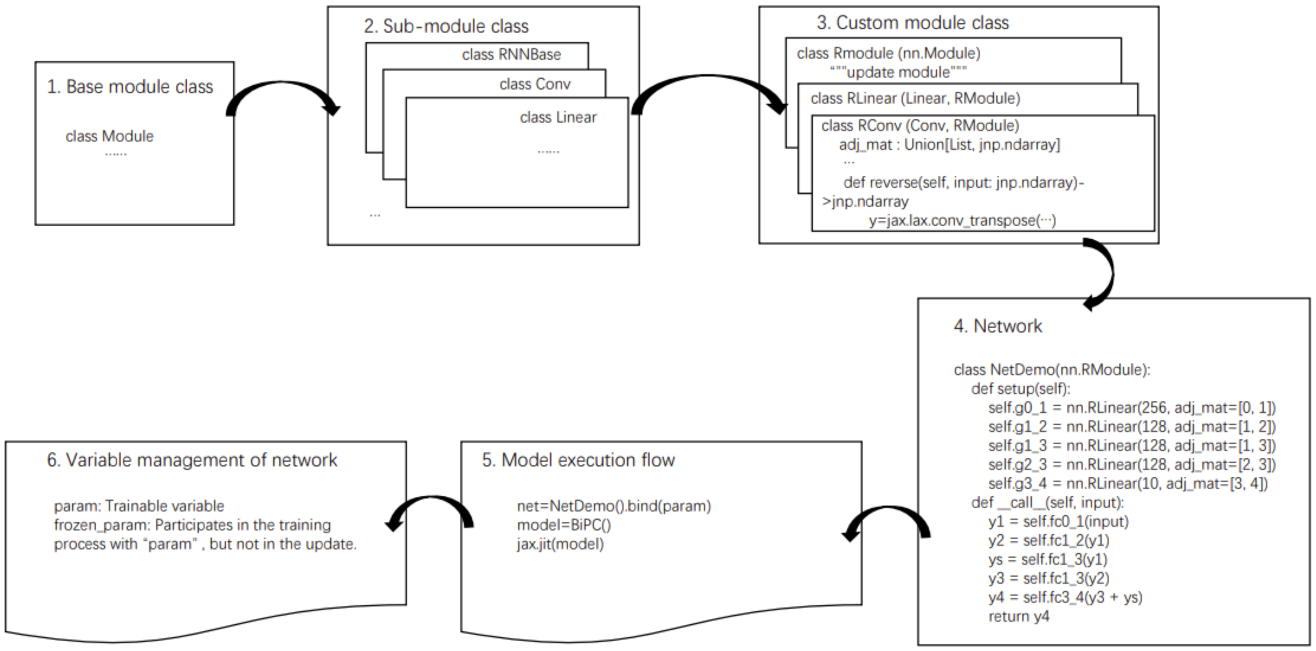

Figure S1. Workflow for network model creation and execution. 1.Base module class. `Module` is the base class that defines the basic structure and behavior of a neural network module, from which other more specific neural network modules can inherit. 2. Submodule class. Submodule class provides the necessary edge operators for defining the network. 3.Custom module class. `RModule` is a pre-defined base class for users to define a bidirectional network. `RConv` and `RLinear` are pre-defined bidirectional operators designed for user convenience. 4.Network. A network is defined by an edge operator with an adjacency matrix property. 5.Model execution flow. The model execution flow involves network instantiation, training mode selection, and model training. Just-in-time (JIT) compilation (`jax.jit`) is applied during model training to accelerate training.

2 THE JAX BACKEND'S ENERGY-BASED FRAMEWORK FOR MODEL EXECUTION

Once the network is created, users can select from various training modes, including hierarchical PC, BiPC, equilibrium propagation, or backprop. A network with an assigned training mode is termed a model. Algorithm 1 outlines the pseudocode for the BiPC model. By utilizing Jax's energy-based framework, JIT compilation can be employed to expedite model training (Listing 1). This process is facilitated by the `jax.jit` function, which compiles both non-gradient computations such as feedforward and feedback operations, as well as gradient-based parameter updates. JIT traces the model's computational graph and compiles it into a static structure. The compilation process includes global optimizations such as operation fusion, constant folding, and memory reuse. The cached computational graph enables the reuse of compiled code in subsequent calculations, improving efficiency. As a result, the initial model compilation is time-intensive, prolonging the first epoch of training.

```
# network instantiation
net = NetDemo()
param = net.init(input=jnp.ones([128, 32, 32, 3]))
out = net.run
```

```
net = net.bind(param)
train_loader = get_train_loader()
test_loader = get_test_loader()

#training mode selection (bidirectional predictive coding)
model = BiPC(loss_f=nn.softmax_cross_entropy, loss_f_b=nn.mse_loss, acc_f=accuracy, n=200, mode=PCMode.SWEIGHT_BISTRICT)
optim = Lalr(eta=1e-4, measure="max", gradient_get_method="harmonic_average")
opt_state = optim.init(param["param"])

# JIT compilation
# add jit for predict and get_grads function.
predict_fn = jax.jit(model.predict, static_argnums=(0,))
get_grads_fn = jax.jit(model.get_grads, static_argnums=(0,))

for i in range(100):
    train_loss_list, train_acc_list = [], []
    for _, (data, label) in enumerate(train_loader):
      start_time = time.time()
      grads, train_loss, train_acc = get_grads_fn(
        net, param, batch_data=(data, jnn.one_hot(label, 10)))
      updates, opt_state = optim.update(grads["param"], opt_state, param["param"])
      param["param"] = apply_updates(param["param"], updates)
      end_time = time.time()
      print("{} {}/{}: train_acc:{:.4f} \t train_loss:{:.4f} \t train_time:{:.4f}".format(
        datetime.now(), i, _, train_acc, train_loss, (end_time - start_time)))
    test_loss_list, test_acc_list = [], []
    test_start_time = time.time()
for _, (data, label) in enumerate(test_loader):
    test_loss, test_acc = predict_fn(net, param, (data, jnn.one_hot(label, 10)))
    test_loss_list.append(test_loss)
    test_acc_list.append(test_acc)
test_end_time = time.time()
```

Listing 1. Example execution of the model

Algorithm 1: Bidirectional predictive coding model

Given : data x, label y, adjacent matrix adj_mat, feedforward lossfun L, feedback lossfun L^b, inference phase lr η_v, learning phase lr η_θ, Initial energyF_{pre}, convergence threshold $threshold$, max iterations max_iters

$L \leftarrow adj\_mat.shape(0)$;
$v_0 \leftarrow x$;
$v_j \leftarrow net.init(adj\_mat, v_0)$; /* Initialize node state */
$v_L \leftarrow y$;
while $\Delta F > threshold$ *or* $n < max\_iters$ **do**
    for $(i, j, g_{i,j}) \in adj\_mat$ **do**
        $\hat{v}_j^f \leftarrow g_{ij}(v_i, \theta_{ij})$;
        if $j == L$ **then**
            $C_f \leftarrow L_f(\hat{v}_j^f, v_L)$;
        else
            $\epsilon_j^f \leftarrow v_j - \hat{v}_j^f$;
        end
    end
    for $(r, j, z_{r,j}) \in adj\_mat$ **do**
        $\hat{v}_j^b \leftarrow z_{rj}(v_r, \theta_{rj})$;
        if $j == 0$ **then**
            $C_b \leftarrow L_b(\hat{v}_j^b, v_0)$;
        else
            $\epsilon_j^b \leftarrow v_j - \hat{v}_j^b$;
        end
    end
    $F \leftarrow SUM[(\epsilon_j^f + \epsilon_j^b) ** 2] + C_f + C_b$; /* Calculate energy */
    for $j \in adj\_mat$ **do**
        $dv_j \leftarrow \frac{\partial F}{\partial v_j}$; /* inference phase */
        $v_j \leftarrow v_j - \eta_v dv_j$;
    end
    $\Delta F \leftarrow ABS(F - F_{pre})$;
    $F_{pre} \leftarrow F$;
    $n \leftarrow n + 1$;
end
for $(i, j) \in adj\_mat$ **do**
    $d\theta_{ij} \leftarrow \frac{\partial F}{\partial \theta_{ij}}$; /* learning phase */
    $\theta_{ij} \leftarrow \theta_{ij} - \eta_\theta d\theta_{ij}$;
end
